# Effectiveness of Theory-Based Physical Activity and Nutrition Interventions in Aging Latino Adults: A Scoping Review

**DOI:** 10.3390/nu15122792

**Published:** 2023-06-18

**Authors:** Ana Maafs-Rodríguez, Sara C. Folta

**Affiliations:** Gerald J. and Dorothy R. Friedman School of Nutrition Science and Policy, Tufts University, Boston, MA 02111, USA

**Keywords:** latinos, aging adults, healthy aging, interventions, theory-based, cultural adaptation

## Abstract

In the United States (US), Latino individuals older than 50 years face health disparities compared to their White counterparts. Considering the rising life expectancy and the projected increase of older Latino adults in the US, this scoping review aimed to determine the effectiveness of theory-based and culturally relevant strategies that promote healthy aging in Latinos. Web of Science and PubMed databases were searched between December 2022 and February 2023 for peer-reviewed articles assessing healthy aging interventions tailored to community-dwelling aging Latino adults. We included nine studies describing the effects of seven interventions on physical activity- or nutrition-related outcomes. Although not always statistically significant, interventions had a beneficial impact on well-being indicators. The most commonly used behavioral theories were Social Cognitive Theory and Attribution Theory. Latino cultural elements in these studies included partnering with community organizations that serve Latinos (such as Catholic churches), delivery of in-person bilingual group sessions by trusted community members (such as *promotoras* or Latino dance instructors), and incorporating values such as family and religion into the health curriculum, among others. Future strategies that promote healthy aging in Latino adults should proactively culturally adapt the theoretical foundations and the design, recruitment, and implementation processes to ensure their relevance and effectiveness.

## 1. Introduction

Life expectancy has increased globally, and the proportion of people older than 60 years is rising at an unprecedented pace [1,2]. This is also the case in the United States (US), where it is estimated that by 2040 21.6% of the population will be 65 and older [3,4,5]. Of those, 34% will be from a racial or ethnic minority, compared to 24% in 2019 [3]. In addition, the projected increase of Hispanic older adults is 161%, greater than for any other racial or ethnic group: 102% for Asian Americans, 80% for non-Hispanic African Americans, 67% for American Indian and Alaska Natives, and 29% for non-Hispanic Whites [3].

The aging process is characterized by progressive physical, cognitive, and psychosocial decline [2,4]. Along with this loss of capacity, aging individuals often accumulate multiple health problems. They are also vulnerable to the emergence of complex health states, such as dementia, frailty, and other geriatric syndromes [2,4,6]. In addition, as people age, they are more likely to be nutritionally vulnerable, as they might have reduced dietary intake and develop nutrient deficiencies [7,8]. As a result, healthcare utilization by this age group is disproportionately high [4,7].

Racial and ethnic health disparities exist in individuals aged 50 and older [9]. For example, a study by Odlum et al. looked at the 20-year trends of poor health indicators among Black, Hispanic, and White and older adults (≥45) [10]. While some trends have improved in the last two decades, Hispanics had the lowest rank for access to healthcare and the worst perception of their general health compared to other racial and ethnic groups [10]. Compared to their White counterparts, Hispanic middle-aged and older adults have a higher prevalence of diabetes, hypertension, and uninsured status [9,10,11]. A recent study using a nationally representative longitudinal survey of US men and women (51 and older) showed that Hispanic older adults had higher depressive symptoms than their counterparts from other races and ethnicities [12]. These trends may be explained by the group’s higher prevalence of mental health problems, physical inactivity, reduced appetite and dietary intake, low solid food consumption, and lower diet quality, among other factors [8,10,13]. A study by Reich et al. suggests that specific ethnic groups might have varying definitions of ‘successful aging’ [14]. For instance, Latinos mention social engagement, positive attitude, and spirituality as important values during the aging process [14]. These differences might also explain why Hispanics in the US rank low in healthcare access and why some campaigns and interventions have not succeeded in sustaining long-term behavior change among Latinos [10,14,15].

In 2020, the United Nations General Assembly declared 2021–2030 as the Decade of Healthy Aging, recognizing the need for effective strategies that promote a long and healthy life for older people [16]. The World Health Organization defines healthy aging as the process of maintaining and improving physical health, mental health, independence, and quality of life [17]. Multiple factors contribute to a successful aging process, mainly by reducing or delaying the risk of developing chronic diseases, nutritional deficiencies, and frailty syndromes [18]. For instance, higher diet quality and healthier dietary patterns have been positively associated with higher cognitive and physical function, as well as better physical and mental health outcomes in older adults [19,20]. Consuming healthy dietary patterns—with adequate amounts of key nutrients such as protein, calcium, vitamin D, fiber, and fluid intake—might aid in diminishing the degenerative alterations that occur during aging, such as cognitive decline or loss of skeletal muscle [21,22]. Increased physical activity (PA) and reduced sedentary time also contribute to the prevention of chronic diseases throughout the lifespan [23].

Many multi-component lifestyle interventions have been developed to promote healthy aging behaviors for older adults or to reduce the risk of developing complications from existing health conditions, such as stroke [23,24,25,26]. This is particularly relevant for community-dwelling adults who face the changes associated with aging and who aim to live independently for as long as possible [27]. Behavioral change techniques or theories have been used increasingly to inform the development of these strategies and to understand how interventions may promote healthy behaviors [23,28]. Previous reviews and meta-analyses have been conducted to evaluate the effect that some of these lifestyle strategies and interventions have had on PA and nutrition-related outcomes among aging and older adults [18,19,23,26,29]. Similar reviews have also focused on the effect of such interventions on vulnerable adults and adults from diverse ethnicities [30,31].

Considering population trends and the ethnic disparities in health indicators among Latinos in the US, it is necessary to identify culturally sensitive and effective strategies that promote healthy lifestyle behaviors for successful aging in this population [10]. To our knowledge, no previous reviews have been conducted to evaluate the effect of lifestyle interventions focused solely on Latino older adults, considering the behavioral or psychological theories used to develop them. The present scoping review addresses this gap by assessing the effectiveness of theory-based interventions among aging community-dwelling Latino adults on PA and nutrition-related outcomes. The term “Latinos” is used in the present work to describe individuals of all genders from Latin American countries or with Latin American heritage.

## 2. Methods

We conducted a scoping review to capture the different approaches and analytical methods that have been used to assess interventions for our population of interest. This type of review adheres to PRISMA guidelines for systematic reviews. We chose to conduct a scoping review since our main goals were to assess the range of evidence and to examine how research related to our topic of interest has been conducted [32,33]. Furthermore, we were interested in identifying and discussing the characteristics, concepts, and practices reported in papers about healthy aging interventions for Latino older adults [32]. A literature search was conducted in the Web of Science and PubMed databases between December 2022 and February 2023 to identify relevant articles published in the previous ten years. Search strings included terms related to nutrition (e.g., diet, nutrition, well-being, lifestyle, eating, food choice); physical activity (e.g., activity, movement, sedentary, exercise, physical activity); theory-based (e.g., theory, framework, behavior change, model, construct); interventions (e.g., treatment, therapy, promotion, education, intervention, program); Latino populations (Hispanic, Latino, Chicano, Mexican-American, Caribbean, Central American, Non-Caribbean, ethnic minority, vulnerable); and older adults (older adult, senior, elder, geriatric, veteran, aging, retired). Search strategies were developed under the guidance of a reference librarian. Table 1 includes the search strings used for the literature search on each database. Additional searches were conducted to identify relevant studies from reference lists.

Articles were eligible to be included in the present review if they (1) reported on a theory-informed intervention or program focused on nutritional or PA behaviors or outcomes, (2) were focused on community-dwelling aging adults ≥50 years old, (3) were developed or conducted among Latino populations (including those that were prioritizing multiple and heterogeneous minority ethnicities), (4) were published in peer-reviewed journals, and (5) were published in English or Spanish.

Articles were excluded if they (1) described interventions or programs with other health behaviors or indicators as primary outcomes [i.e., smoking cessation, adherence to medical treatment] or without specifying any theoretical foundation, (2) were not focused on Latinos (e.g., studies that were conducted with individuals from Spain), (3) included individuals <50 years old, (4) were observational, development or methods studies, (5) were conference abstracts, data sets or book chapters, (6) did not have a full text available, (7) were not available in English or Spanish, or (8) did not answer the research question.

Searches resulted in 663 articles, and results were exported into Mendeley. All duplicates were removed. The articles were screened by one person (AM), and this was performed on title, abstract, or full text, based on inclusion and exclusion criteria. Data extraction from the articles was performed using Microsoft Excel. It included citation, country, name of the intervention/trial (or short description), study design, duration of intervention, sample size and participants’ characteristics, inclusion criteria, aim, setting and mode of delivery, intervention description, comparison group (if applicable), theoretical foundations, main outcomes and measurements, key results, strengths, limitations, and culturally sensitive characteristics for Latinos. If available, protocol or paper methods were consulted to obtain additional details about the interventions’ development.

## 3. Results

**Figure 1:**
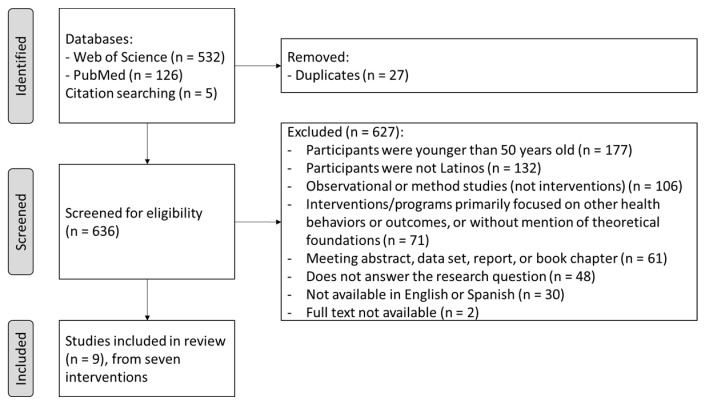
Flowchart of study selection process.

Considering the inclusion and exclusion criteria, a total of nine studies were included in this review, assessing the effect of seven interventions [9,15,34,35,36,37,38,39,40]. Figure 1 shows the flow chart of the study selection process.

### 3.1. Study and Participant Characteristics

Table 2 shows the studies’ and participants’ characteristics. All studies except one were conducted in the US; the Centre of Social Health Services for Older Adults (COASH) program was conducted in Mexico [34]. Five of the studies were randomized controlled trials (RCT) or cluster-RCT (Let’s Walk!, Worth the Walk, Community of Voices (COV), BAILAMOS^TM^, and BAILA) [36,37,38,39,40]. Three studies had a pre-post quasi-experimental design (COASH, *Abuelas en Acción* (AEA), and Healthy Living Wellness Program (HLWP)) [9,15,34,35]. Two were pilot studies (COASH and BAILAMOS^TM^) [34,39], although the latter evolved and was scaled up to an RCT (BAILA) [40]. Only two studies (AEA and BAILAMOS^TM^) [35,39] incorporated a mixed-methods approach to assess participants’ perceptions of the interventions. Four studies (AEA, Let’s Walk!, Worth the Walk, and BAILA) [35,36,37,40] included a maintenance or follow-up phase ranging from 2 to 20 months after the intervention had concluded. All studies included in this review incorporated partnerships with community centers to different extents. The Worth the Walk study formed four mini-Community Action Boards to co-develop the intervention curriculum for different ethnicities [37,41].

An inclusion criterion of all nine studies was that the participants were community-dwelling adults aged 50 years or older. Sample sizes ranged from 30 to 572 participants (HLWP and Let’s Walk!, respectively) [9,36], and participants’ mean age ranged from 64 to 77 years (BAILA and COASH, respectively) [34,40]. Participants were mostly female in all studies, ranging from 68% (COASH) [34] to 100% (AEA) [15,35]. Three studies recruited participants from diverse ethnic backgrounds, as their interventions were intended for adults from multiple minority groups [9,37,38]. In those studies, the percentage of participants who identified as Latino was 47% for HLWP [9], 27% for Worth the Walk [37]; and 18% for COV [38].

### 3.2. Interventions

Table 3 shows the characteristics of each intervention and the theoretical foundations that were used. All interventions were conducted in partnership with local community senior, social and/or health centers [9,15,34,35,36,37,38,39,40]. For example, COASH was incorporated into a social service complex within a larger hospital in Mexico City [34], and Worth the Walk became part of the regular programming within senior centers [37]. AEA was delivered in Catholic churches [15,35], and both BAILAMOS^TM^ and BAILA relied on churches to gain trust and to recruit community members [39,40].

Four interventions conducted formative research to better understand the context and needs of Latino aging adults. Findings from these strategies were then used to inform the development and implementation of the interventions (AEA, Worth the Walk, COV, and BAILAMOS^TM^ (which were also incorporated into BAILA)) [15,35,37,38,39,40,41,42,43].

All studies included in-person educational sessions (individually or in groups), workshops, discussions, or activities to deliver the respective intervention curriculum and to collect data measurements. Control groups, for the studies that had them, also received in-person education, though with different curricula or delivery strategies. AEA and Worth the Walk additionally incorporated motivational phone calls and activities for participants to do at home [15,35,37]. Different types of trained facilitators delivered the interventions. For example, COASH and HLWP relied on health professionals and interns to deliver the program components [9,34]. The Let’s Walk! intervention used trained health educators from the community and from specific ethnic backgrounds [36], and AEA used *promotoras de salud* (community health leaders) for implementing the education sessions [15,35]. *Promotoras* are frontline female Latino workers who have a unique set of skills, knowledge, and cultural competence that has historically allowed them to improve the health of Latino communities. *Promotoras* have a distinctive identity and roles within existing social networks [44]. COV relied on professional choir directors and accompanists to deliver the program, all of whom completed training on the intervention components [38]. The delivery of Worth the Walk also included trained case managers already working at the senior centers [37]. The dance program of BAILAMOS^TM^ and BAILA was co-created and delivered by a trained professional Latino dancer [39,40]. For the BAILA trial, dance sessions were led by the same professional dancer at the beginning of the trial, and later by trained indigenous participants. The health education component of the control group was delivered by trained research team members [40].

All interventions in this review were multicomponent, yet their content and format varied considerably. The COASH pilot program [34] provided a series of services to Mexican adults affiliated to the Mexican Institute of Social Security (IMSS). Hence the intervention was incorporated into services that were already offered for older adults at a healthcare center. The two main components of the active aging intervention functioned sequentially [34]. First, for each participant, health professionals completed a comprehensive eight-domain geriatric assessment, which included clinical history, nutritional status, psychological health, socioenvironmental information, and lifestyle assessment (which considers food consumption, smoking, alcohol intake and PA), among others. Secondly, health professionals used this information to determine which active aging services participants had to attend (services included required, selective, and optional components) [34]. These active aging services covered topics related to healthy aging (nutritional counseling, social therapy, physical and occupational therapy, self-care, and leisure time, among others), and were mostly delivered in group sessions weekly, every two weeks, or monthly [34]. Similarly to COASH, HLWP also included individual health screening [9,34]. In HLWP, the screening results could be discussed at individual meetings. These weekly assessments took place at the beginning of each in-person meeting and were followed by guided education sessions [9]. The educational components of HLWP were also delivered by health professionals, in this case Registered Nurses. However, a noteworthy difference between COASH and HLWP is that the content of HLWP was more focused on reframing current beliefs and attitudes of enrolled participants to encourage them to engage with healthy lifestyle behaviors [9]. The curriculum of HLWP was based on recommendations from Healthy People 2010 and the Seventh Report of the Joint National Committee on Prevention, Detection, Evaluation, and Treatment of High Blood Pressure [9]. The recommended guidelines were delivered through the weekly education sessions, which included topics such as hypertension, diabetes, body image, weight loss, effect of lifestyle modifications on chronic diseases, and understanding lipid values, among others [9].

AEA was the only intervention that targeted female Latino adults exclusively. Although this study was not solely for grandmothers, it was developed as a response to the role that Latino women play in their families, their responsibilities as caregivers of other family members—particularly children—and the effect that those roles and responsibilities have on motivation to remain healthy [15,45]. The role that Catholicism and faith play in the lives of older female Latino adults also informed the AEA curriculum [15,46]. This nutrition, PA, and stress management intervention focused on activities that could be done with children or at home and identified Catholic Saints and prayers that aligned with the behaviors being promoted [15,35]. The intervention included individual meetings, educational workshops, and follow-up phone calls. The content of the workshops included healthy living, healthy eating, getting active, buying healthy food, being active in ‘your’ way, stress management, and overcoming barriers [15,35]. AEA also included a 3-month maintenance period, during which participants received biweekly motivational phone calls [15,35]. Of note, AEA used the RE-AIM (Reach, Effectiveness, Adoption, Implementation, and Maintenance) framework to determine the program’s effectiveness and reach, but also barriers and facilitators associated to its implementation [15,35].

Let’s Walk! and Worth the Walk were both centered around walking [36,37]. In particular, walking was promoted through in-person group education sessions that combined promoting PA with other healthy aging topics [36,37]. Of note, Worth the Walk built on some components from Let’s Walk!, which in turn included some modified elements from the EnhanceFitness^®^ program (developed by the University of Washington in partnership with Sound Generations and Group Health Cooperative, in Seattle, WA, USA) [36,41,47,48]. In both studies, participants received pedometers and were exposed to health education sessions for a month. However, Let’s Walk! focused on improving PA-related indicators and behaviors, including physical and mental quality of life, exclusively of Latinos [36], while Worth the Walk aimed to increase stroke knowledge among four different minority groups. Hence, the Worth the Walk’s intervention curriculum, delivered through double the number of sessions than in Let’s Walk!, included stroke risk factor education elements (such as blood pressure, strategies to cope with stress, and communication with doctors, among others), based on culturally adapted materials from the American Heart Association/American Stroke Association [37]. Another relevant difference between Let’s Walk! and Worth the Walk was that Worth the Walk was integrated into existing programming at the partnered senior centers (similar to COASH), so the education sessions were delivered by trained case managers from those centers, as mentioned above [37,41]. One more difference is that Worth the Walk had a delayed-intervention control group [37,41].

COV was a unique intervention: it combined promotion of healthy aging and well-being with choir classes for racially and ethnically diverse adults [38]. This study had a 1-year duration, during which participants received 44 choir sessions and attended public performances. Trained choir directors and accompanists delivered the singing sessions that explicitly incorporated cognitive, physical, and psychosocial techniques to encourage participants to engage in healthy aging-related behaviors [38,42]. Examples include changing seating arrangements, adding choreography to the songs, practicing listening to other singers, focusing on breathing techniques, including time for reflection, and participating in a 10 min break for refreshments and socialization [42]. To make the intervention culturally relevant for the different ethnicities of the participants, music and songs were tailored to their preferences and aligned with their backgrounds. Despite this tailoring, this study had the lowest percentage of Latino adults [38].

Finally, BAILAMOS^TM^ and BAILA delivered the same 4-month dance program to older Latino adults [39,40] to assess its effect on PA levels. Four Latino dance styles were taught through one discussion meeting and several instruction sessions to learn the dance moves [39,43]. These dance styles were selected based on their relevance to Latino culture and their moderate intensity. During the discussion sessions, information from the 2008 PA Guidelines for Americans was covered [43]. The program also included *Fiestas de Baile* [dancing parties], to which participants were encouraged to bring food and drinks to share [43]. The intervention included providing an accelerometer to all participants, even those in the control group (who received health education instead of dance classes) [39,40]. The health education curriculum for the control group differed from the pilot to the trial study in several ways. For example, in the BAILAMOS^TM^ pilot study topics included stress, home safety, nutrition, dealing with chronic diseases, healthy relationships, and boosting the memory, among other topics [39]. In the BAILA trial, sessions for the control group included additional nutrition-related topics, such as My(food) Pyramid, and reading food labels [40]. Slight modifications were made from one delivery to the other: BAILA also included a 4-month maintenance phase with additional and explicit strategies to facilitate adoption and maintenance of active behaviors [39,40]. In particular, BAILA incorporated a train-the-trainer model [40], an effective and cost-efficient education model for scaling up or implementing interventions through members of an organization or community [49].

**Table 3:** Interventions’ details.

Intervention Name	Setting and Mode of Delivery	Intervention Description	Comparison	Behavioral Theory Foundations
Centre of Social Health Services for Older Adults, (COASH) pilot program [34]	Setting: Social service complex within hospital in Mexico City. Delivery: In-person provision of active aging services (including individual and group sessions) provided weekly, every two weeks or monthly. Delivered by trained multidisciplinary health professionals working at the center.	Provision of two services for older adults: (1) Comprehensive Geriatric Assessment (CGA): This included (A) Clinical history, laboratory tests, health diagnoses; (B) Nutritional status (mini-nutritional assessment and BMI); (C) Oral health; (D) Functional status; (E) Psychological health; (F) Podiatric assessment; (G) Lifestyle assessment (e.g., food consumption, smoking, PA); and (H) Socioenvironmental information. (2) Active Aging: - Required components (all participants needed to attend these): Social therapy, physical therapy sessions, and occupational therapy sessions. - Selective components (only some participants needed to attend these, based on CGA): Mental health (psychotherapy and cognitive therapy) and self-care sessions (nutritional counseling and occupational therapy). - Optional components (free choice to attend): Leisure time and communication technology sessions.	Pre-intervention measures.	Cognitive behavioral techniques to facilitate the emergence and maintenance of desirable behaviors: positive communication, verbal incitement, reinforcement, motivation, and problem resolution, among others.
*Abuelas en* *Acción* (AEA) [Grandmothers in Action] [15,35]	Setting: Catholic church facilities. Delivery: - Monthly in-person one-on-one meetings and group workshops. - Weekly and biweekly follow-up phone calls. Delivered by trained *promotoras* (community health leaders) from the community.	Culturally sensitive behavioral change curriculum delivered in three IG: - Traditional: Three components: (A) individual meetings; (B) six educational workshops (Introduction to healthy living, Healthy eating, Get active, Buying healthy food, Be active your way, Stress management and overcoming barriers); and (C) weekly follow up motivational phone calls (biweekly during maintenance phase). - Intergenerational: Same components than traditional, with the addition of family activities into the curriculum (e.g., incorporating grandchildren into workshops, handouts for activities to conduct while caring for grandchildren). - Religious: Same components than traditional, with the addition of religious content integrated into the curriculum (e.g., workshops themes paired with a prominent Catholic Saint that illustrated the behavior, prayers, scripture reading).	Comparisons across time points.	Socio-Ecological Model. Transtheoretical Model. Social Cognitive Theory.
Healthy Living Wellness Program (HLWP) [9]	Setting: Senior citizen housing project community centers. Delivery: In-person weekly education and exercise sessions (including individual and group activities). Delivered by Registered Nurses and student interns fluent in Spanish and English.	Healthy living and exercise curriculum developed based on Healthy People 2010 and the Seventh Joint National Committee of Prevention, Detection, Evaluation, and Treatment of High Blood Pressure. Weekly sessions were structured as follows: - Health screening assessment: Individual revision with each participant. - 30 min guided exercise session: Using videos, music, dancing, chair yoga, exercise cardio videos, and walking. - Formal education sessions: Topics included hypertension, diabetes, body image and weight loss, effects of lifestyle modifications on hypertension and diabetes, among others. Sessions included guidance for measuring these indicators by themselves. - Informal group sessions: Opportunity to clarify questions and to practice measuring their own indicators.	Pre-intervention Measures.	Roy adaptation model: curriculum was classified into the four adaptive modes: physiologic mode (focused on diet and exercise behaviors), self-concept mode (focused on a better perception of self), role function (promoting self-management), and interdependence mode (facilitating the development of social networks).
Let’s Walk! [¡*Caminemos*!] [36]	Setting: Community-based senior centers. Delivery: In-person weekly education and exercise classes (then monthly and every two months). Delivered by a trained bilingual health educator from the community.	- Core sessions: * Four weekly 1 h group discussion (with 8 to 10 participants) centered around age attribution retraining. * Four weekly 1 h exercise classes targeting muscle strength, endurance, balance, and flexibility. Classes were a modified version of the EnhanceFitness^®^ Program, designed to be safe for seniors and offered both sitting and standing options for each exercise. A *fotonovela* was used during the second half of the intervention. - Reinforcement schedule (after the 4-week core intervention): * For 11 months: participants received eleven monthly 1 h exercise + 1 h education classes * For the last 12 months: participants received six 1 h exercise + 1 h education classes, delivered every two months [47,48]	CG: Exposed the same core and reinforcement schedule as the IG. Group discussions included a generic health education curriculum, with topics around senior wellness. Didactic Power Point presentations were used. Exercise classes were the same.	Self-efficacy (Social Cognitive Theory). Attribution Theory.
Worth the Walk [37]	Setting: Community senior service centers serving Latino, Korean, Chinese, and Black older adults. Delivery: In-person twice-weekly sessions. Delivered by trained bilingual case managers already working at the serving centers.	Eight 1 h intervention sessions held twice weekly for one month. Content included promoting walking and stroke knowledge (disparities in stroke, stroke outcomes, warning signs, blood pressure control to reduce risk burden). Participants were provided with a diary to record their daily PA. They also received a pedometer and telephone reminders to wear it Last two sessions were culturally tailored to each racial/ethnic group to enhance relevance and impact, using insight from formative work (e.g., Latinos sessions were titled ‘Walking is Good for Health and Relieving Stress’, and ‘Family Matters’, while sessions for African Americans were titled ‘Walking is Good for the Body’ and ‘Walking is Good for the Soul’) [37,41].	Delayed intervention. Participants in the CG received the same frequency of contact from research staff (call reminders), as well as the pedometer and same monetary compensation.	Social Cognitive Theory. Attribution Theory.
Community of Voices (COV), [*Comunidad de Voces*] [38]	Setting: Administration-on-Aging-supported senior centers serving racial/ethnically diverse communities. Delivery: In-person weekly choir sessions. Delivered by trained professional choir directors and accompanists (some were bilingual).	Forty-four weekly 90 min choir sessions (and three or four informal public performances), with culturally relevant music styles. Sessions included cognitive, physical, and psychosocial engagement techniques (e.g., sitting and standing, moving to different parts of the room to sing, setting and working toward a common goal, body posture and breathing, focus on abdominal and chest muscles, refreshments and socialization opportunities, discussion of songs and their cultural history, among others) [42].	Delayed intervention, although comparison took place at 6 months.	Translational research approach: evidence on the benefits of singing for older adults (cognitive, physical, and psychosocial engagement components). Self-efficacy and social support.
BAILAMOS^TM^ [We are dancing] Pilot Study [39] BAILA [Dance] trial [40]	Setting: Senior centers (IG) and Catholic churches (CG). Delivery: In-person twice a week dance classes. Delivered by trained Latino dance instructors (IG in BAILAMOS^TM^ and BAILA); and trained research team members (IG in BAILA) and trained health educators (CG in BAILAMOS^TM^ and BAILA).	IG in BAILAMOS^TM^ and BAILA: Dance sessions included (32 total): (A) warm-up and stretching; and (B) instructions of the respective dance style, and steps for singles and couples. Couples learned steps of both leaders and followers and continually rotated partners. Twice a month, participants attended *fiestas de baile* (dance parties) in which they had time to practice the learned steps until that point. These were part of the program. Participants were encouraged to bring food and drinks. IG in BAILA trial: Instructor also emphasized increasing household and transportation PA outside of the program. Monthly discussion sessions took place, done before the dancing session, delivered inperson by a research team member with expertise in PA. For the maintenance phase: Following the “Train-the-Trainer model”, two to three volunteer indigenous leader(s) (participants from the dance condition from each site) were asked about their interest and willingness to become dance instructors. Indigenous leaders across study sites were taught the same additional dance steps to add to existing moves, and they led the original treatment group members at the respective site. All treatment group participants had the opportunity to continue dancing.	CG received weekly 2 h sessions to promote social contact similar to the IG. Topics included stress, home safety, nutrition, dealing with chronic diseases (e.g., diabetes, cancer, and osteoporosis), immunizations, healthy relationships, boosting memory, and making the most of medical appointments. BAILA trial: The curriculum for CG covered additional topics: My (food) Pyramid, and food labels.	Self-efficacy. Social Cognitive Theory.

BMI = Body Mass Index; IG = Intervention Group; CG = Control Group.

### 3.3. Theoretical Foundations

In most studies, the intervention was developed based on a combination of constructs from more than one theory. The most common theory used as a basis for the interventions was Social Cognitive Theory (SCT), used in four studies (AEA, Let’s Walk!, Worth the Walk, and BAILA) [15,35,36,37,40]. Attribution Theory was mentioned in two studies (Let’s Walk! and Worth the Walk) [36,37], in which attribution and retraining techniques were used to encourage people to rethink their beliefs about the controllable and uncontrollable aspects of aging to better promote behavior change around the controllable factors [36,37].

Three studies (COASH, COV, and BAILAMOS^TM^) used behavioral constructs independent of an entire theory [34,38,39]. Self-efficacy was the most common and was emphasized in some studies, in addition to the behavioral theory used [36,38,39,40]. COASH and COV mentioned other cognitive and behavioral techniques used to inform the development or delivery of the programs, such as positive communication, verbal incitement, reinforcement, goal setting, and social support, among others [34,38]. AEA also applied the Socio-Ecological Model (SEM) and the Trans-theoretical Model (TTM) in addition to SCT to design the intervention curriculum and to select implementation strategies [15,35]. HLWP used the Roy Adaptation Model (RAM), which considers aging people as adaptive systems who interact with their environments through different stimuli [9].

There was variation in how each study incorporated (or reported the incorporation of) behavioral theory components into their program content or delivery and evaluation strategies. COASH did not explicitly mention the use of a specific theory, yet it describes how particular cognitive behavioral techniques were incorporated into the intervention modules (required and selective) to encourage behavior change and adherence to the program [34]. The cognitive behavioral strategies used to deliver these modules included progressive involvement in different physical activities (strength training, balance, and flexibility); self-care tasks; use of computers, mobiles and internet; and nutritional management of chronic diseases, among others [34]. The progressive nature of the sessions aimed to facilitate mastery and self-efficacy regarding the different activities. The trained health professionals also used other strategies to promote behavior change when implementing the modules and interacting with participants. These strategies included positive reinforcement; promotion of motivation, self-evaluation, self-motivation, and self-effort; encouraging resolution of problems; and positive communication and verbal persuasion, among others [34].

AEA used three theories to inform its development and implementation [15,35]. For example, the SEM was used as a framework to guide the formative research, particularly the interviews conducted with older women from the community. SEMs help explain the roles of multiple levels of influence in shaping behavior, as a result of the interaction between an individual and the context in which they live [15]. The researchers were interested in understanding the complex and relevant socio-cultural and environmental factors influencing older Latino women’s behaviors and beliefs [15]. One way the SEM was explicitly incorporated into the formative work in AEA was through photo-elicitation. For this, participants were given a disposable camera and asked to take pictures of salient and personally meaningful features in their lives, such as people, places, and activities. This technique allowed the researchers to identify familial, environmental, and cultural aspects that facilitate or hinder Latino older women’s efforts to adopt healthier lifestyles [15]. Consequently, faith and caregiving roles were incorporated as central aspects of the AEA curriculum. For the delivery of the program, AEA used elements from TTM and SCT [35]. The TTM considers five stages of change through which individuals progress when changing behavior. Individuals advance through these stages by means of ten fundamental processes of change and by weighing the pros and cons of change (decisional balance) [35,50]. In contrast, the SCT considers people’s abilities to interact, alter and construct environments that align with their purposes [35]. SCT posits that efficacy beliefs (belief that one can accomplish a behavior) and outcomes expectancies (belief that engaging in the behavior will yield positive results) are key determinants that influence motivation and behavior [36,51]. In AEA, TTM’s processes of change were integrated via individual meetings and motivational phone calls with participants. During the training of *promotoras* who delivered the intervention, one of the nine modules included an overview of readiness for change, according to the stages of change from TTM [35]. Furthermore, SCT principles were incorporated into the workshops by providing participants with knowledge, skills, and experiences around PA, nutrition, and stress-management behaviors [15,35].

HLWP used RAM as a guiding framework for its development and assessment. This model was selected based on the concept that adaptation to aging is a complex process, particularly because individuals have to adjust to changes in their internal and external environments [9]. According to RAM, after an adaptive system (in this case, aging adults) encounters stimuli and implements coping activities, resulting behavior change can be observed in four adaptive modes (physiologic, self-concept, role, and interdependence) [9,52]. The promotion of adaptation (as opposed to maladaptation) in each mode contributes to health and better quality of life, which makes this a particularly relevant model for understanding the aging process [9,52]. Hence, the curriculum of HLWP was classified into four categories (corresponding to the four modes from RAM): physiologic mode (focused on diet and exercise behaviors), self-concept mode (focused on a better perception of self), role function (promoting self-management), and interdependence mode (facilitating the development of social networks) [9]. HLWP also used RAM as a framework to assess and report study outcomes. For example, Body Mass Index (BMI) and anthropometric measures (circumferences) were grouped under the physiologic mode, while the Mood and Feelings Scale was reported under the self-concept mode [9].

Let’s Walk! and Worth the Walk used a combination of Attribution Theory and SCT, in different capacities, to promote PA [36,37]. Attribution Theory posits that individuals explain their successes or failures based on causal attributions [47]. These can be classified into three dimensions: internal or external locus of causality, stability (fixed or changeable), and controllability [36,47]. Individuals are more likely to change their behavior when they believe that outcomes are malleable and within their control [36,47].

Let’s Walk! used Attribution Theory as the key guiding framework for developing, delivering, and assessing the intervention, along with some components from SCT, particularly around self-efficacy [36]. The authors describe Let’s Walk! as an Attribution Retraining Intervention, centered around changing the belief (attribution) that physical inactivity is an expected part of aging. Curriculum facilitators were trained on relevant strategies from Attribution Theory and SCT [36]. Hence, the core intervention promoted age-reattribution. During the early sessions participants identified reasons for not being active, which were then classified into the attribution domains. During the four weeks of the program, as a group, participants in the intervention arm were exposed to the attribution retraining of mutable and controllable factors [36,47,48]. As for SCT, the intervention explicitly incorporated strategies to enhance participants’ self-efficacy and outcome expectations for PA. Such strategies included training the facilitators to provide verbal persuasion to participants, modeling exercises and movements during the classes (for vicarious experience), establishing clear goals and action plans (to identify performance accomplishment), and opportunities to reflect on attitudinal changes around PA [36]. Additionally, Let’s Walk! included these behavioral foundations in its secondary outcomes, as it assessed participants’ expectations regarding aging, self-efficacy for exercise, and outcome expectations for exercise [36].

Worth the Walk was very similarly based on SCT and Attribution Theory, with slightly less emphasis on the latter, and incorporated other SCT constructs (such as knowledge and social norms) more explicitly [37,41]. Researchers found that racial and ethnic minorities knew less about stroke than their non-Latino White counterparts, and, therefore, knowledge (provided in a relevant manner and attuned to social and cultural norms) was actively incorporated into the program curriculum [37]. The curriculum for Worth the Walk provided knowledge about stroke and also included specific strategies to promote self-efficacy for stroke preparedness. Participants received a diary to record their daily PA, with the intention that this would foster performance accomplishment and internal positive reinforcement [41]. This study also assessed these behavioral components in the form of questions to evaluate knowledge about risk of stroke, self-efficacy for conducting the tasks and activities related to managing stroke risk, and outcome expectations, among others [37,41].

COV incorporated theoretical foundations into activities to deliver the content of the choir program [38,42]. For example, the authors described integrating cognitive, physical, and psychosocial components to promote engagement with the intervention as hypothesized mechanisms of action for the program [38]. Physical components aimed to promote PA in participants, and included strategies such as sitting and standing, moving around the room, and focusing on posture and breathing, among others [42]. The cognitive and psychosocial components promoted self-efficacy (around singing and PA) and emotional and informational support. Some techniques to achieve this included aural learning of separate parts of the songs, singing from written song lyrics or musical notation, listening to fellow singers, reviewing previously learned songs, setting a common goal to work towards, opportunities for socialization (with refreshments), and discussions of songs and their cultural meaning, among others [42]. Moreover, the COV study protocol included tools intended to assess the program’s effect on these behavioral constructs, in specific social support and self-efficacy (around mastering new skills with practice over time) [42].

BAILAMOS^TM^ and BAILA used SCT as a guiding theoretical framework [39,40]. In particular, the dance program protocol identified self-efficacy as a key underlying mechanism influencing PA and health outcomes [43]. As such, it was incorporated as both a mediator and as an outcome in the protocol hypotheses [43]. Additionally, the dance program curriculum was centered around increasing self-efficacy. Strategies included sequencing the dance styles by level of difficulty (starting with the easiest one) to ensure that participants had mastery accomplishment. Vicarious experiences were also incorporated via social modeling by other participants and the dance instructor, who also provided verbal encouragement [39,43]. The dance program protocol detailed different tools to measure self-efficacy, particularly around lifestyle, exercise barriers, gait, and balance [43]. Of note, after BAILAMOS^TM^ [39] evolved into BAILA [40], other SCT components were more explicitly incorporated into the group discussions, such as increasing knowledge, outcome expectations and social support (in addition to self-efficacy) [40,43]. Lastly, BAILA used the Behavior Change Consortium model of treatment fidelity to monitor the intervention implementation [40].

### 3.4. Outcomes and Results

The main outcomes of the studies included in this review varied, although they were all centered around healthy aging and overall wellness. All studies included a PA or nutrition-related measure as a primary or secondary outcome, mostly by a combination of self-reported and objective measures (for example, through the use of pedometers, accelerometers, anthropometric measures or biomarkers). However, the measurement of these outcomes was inconsistent across studies. Overall, while not all effects were statistically significant, these studies generally showed a positive trend in healthy aging related outcomes. Furthermore, studies that included a qualitative component found that participants valued the interventions as an opportunity to connect with others, to reflect on their health, and to feel better [9,15,35,39]. Table 4 includes a summary of relevant outcomes, measurements, and key findings of each study.

#### 3.4.1. Physical Activity Related Outcomes and Results

PA was the most consistently reported outcome, and four studies measured it as change in weekly minutes of PA. AEA (which had a small sample size) found a non-statistically significant increase in weekly minutes of PA and in minutes of activity bouts when comparing different study timepoints [35]. HLWP reported an increase in PA after the intervention (compared to baseline values) and in the percentage of participants who reported exercising daily at the end of the intervention (73%), but without statistical details nor comparison to baseline values [9]. BAILAMOS^TM^ and BAILA also found a higher increase in weekly minutes of PA in the intervention group (IG) compared to the control group (CG), but this difference was only statistically significant in BAILA, which had a larger sample size [39,40]. On the other hand, BAILAMOS ^TM^ found statistically significant differences in increased self-reported PA in leisure time between groups, which was also the case in BAILA [39,40]. The latter study did not find statistically significant differences in household minutes of PA between groups [40]. Let’s Walk! and Worth the Walk both measured daily or weekly steps determined by a pedometer [36,37]. Let’s Walk! included self-reported PA evaluated using the Yale Physical Activity Survey (YPAS) [36,37]. Let’s Walk! found a statistically significant increase in weekly steps among participants in the IG compared to those in the CG between 1 and 12 months. While scores from the YPAS were higher in participants from the IG (which suggests more total time spent on different activities in a typical week), these differences were not statistically significant [36]. In Worth the Walk, researchers found that participants in the IG had more daily steps at 1 and 3 months of the study than participants from the CG [37]. The authors note that even though effects on this particular outcome are statistically significant, they may not have clinical significance [37].

COV reported changes in body strength, balance and walking speed. No statistically significant differences were found between groups, but the authors reported difficulty in measuring some of their outcomes [38]. COASH measured similar outcomes [34]. For example, authors reported statistically significant improvements in overall mobility score (which considers gait and balance) and in occupational functioning score (one of the sub-scores assesses motor skills) at the end of the intervention, compared to baseline values [34]. Non-statistically significant changes were found in participants’ independence doing basic activities (some of which include PA components, such as walking around the house) [34]. COASH also measured the change in the proportion of participants who complied with the PA Guidelines of engaging in at least 150 min/week of moderate to vigorous activities. The authors found a statistically significant increase in the proportion of participants who were active after the intervention [34]. Similarly, AEA reported a non-statistically significant increase in participants who could be classified as active at the end of the intervention [35]. In addition, AEA’s qualitative evaluation revealed that participants felt more motivated to walk more and found walking and resistance training to be interesting activities. Participants also shared their experience using homemade weights to exercise at home, and found creative ways to be more active around their houses, such as increasing movement by playing music while cooking [35].

#### 3.4.2. Nutrition Related Outcomes and Results

There was substantial variability in how studies measured and reported nutrition-related behaviors and outcomes. AEA was the only study that reported nutrition-related outcomes in detail. These were measured using a Food Frequency Questionnaire and 24 h recalls as well as open-ended questions that asked participants for their perceptions around healthy eating components of the program [15,35]. Across different time points there were statistically significant and favorable intervention effects on the percentage of participants who ate three meals per day, the number of days per week that vegetables and fruits are consumed, and the number of fried foods eaten in a day [15,35]. Other nutrition-related outcomes and behaviors did not have statistically significant changes: number of meals per day; hours between breakfast and lunch, and between lunch and dinner; and number of vegetables and fruits consumed per day [15,35]. Additionally, in response to open-ended questions, participants described their increased awareness of healthy dietary behaviors (such as increasing their fiber intake through fruits, vegetables and whole grains; reducing their fat intake by using less oil during cooking, substituting animal fat (as lard) with vegetable oil, buying 2% milk instead of whole milk, or avoiding traditional foods that are high in fat, such as *enchiladas*; and reading food labels when buying canned or packed products) [35]. Participants in AEA mentioned feeling more confident in talking about diet with their families and making efforts to improve their own and their families’ food choices [35]. Of note, some participants (n = 6, out of 14 interviewed) reported guilt after eating, or not feeling ready to eliminate fatty foods (such as tacos, tortillas, or fried meat), from their diet, as these allowed them to maintain cultural ties with their countries of origin [35]. COASH included some nutrition-related behaviors in two of its assessment tools: the Barthel index (which assesses levels of dependency) includes eating as one of the ten basic activities for daily living, and the Lawton and Brody scale (which similarly considers levels of dependency) includes preparing food and shopping as two of the eight instrumental activities of daily living [34]. While participants reported improvement in these areas, the pre-post changes were not statistically significant [34]. HLWP briefly assessed the program’s effect of the program on healthy eating using an end-of-program questionnaire that asked participants to report if they were complying with daily recommended dietary modifications, to which 83% of participants replied affirmatively [9]. The other studies did not specifically incorporate nutrition-related outcomes. However, during the cluster randomization process of the COV study, the percentage of individuals with nutrition risk was considered when balancing center characteristics across experimental groups [42].

#### 3.4.3. Other Health Related Outcomes and Results

Three studies measured other health indicators, including anthropometric (BMI, waist circumference) or clinical measures (blood pressure), biomarkers (blood glucose), and metabolic indicators (change in energy expenditure). HLWP included the greatest number of these outcomes, followed by Worth the Walk. In HLWP, researchers collected data on body weight, BMI (measured using an electronic scale and a wall measuring stick), waist circumference (measured using a tape measure), blood pressure (measured using an electronic blood pressure monitor), and blood glucose (which was self-reported) [9]. Despite the small sample size, statistically significant decreases were found in BMI, waist circumference, and blood glucose [9]. The authors report a non-statistically significant decrease in weight and blood pressure [9]. Worth the Walk also measured BMI, blood pressure and biomarkers, in particular, non-high-density lipoprotein (non-HDL) cholesterol, C-reactive protein, and glycated hemoglobin (HbA1c) [37]. Changes in these indicators were not statistically significant [37]. Finally, BAILAMOS^TM^ assessed the cardiorespiratory fitness of participants using a previously validated equation without exercise testing which takes into account participants’ sex, age, BMI, resting heart rate, and PA level (on a scale from 1 to 5) [39]. Cardiorespiratory fitness did not change in either group [39].

#### 3.4.4. Well-Being Related Outcomes and Results

Five studies assessed their interventions’ and programs’ effect on other well-being outcomes. For example, occupational functioning and perception of health-related quality of life was assessed in COASH [34]. Emotional well-being, moods, feelings, and attitudes towards life were assessed in AEA, HLWP, COV, and BAILAMOS^TM^ [9,15,35,38,39]. In COASH, there was a statistically significant improvement in participants’ occupational functioning scores (which considers skills needed for communication and interaction, as well as motor and environmental skills) [34]. Changes in perception of quality of life were also statistically significant [34]. This indicator was measured using the WHOQOLBREF tool, which asks a person to consider their physical and physiological health, as well as their social relationships and environment [34].

AEA measured depressive symptoms and emotional well-being using a validated tool and open-ended questions [15,35]. At the end of the program there was a statistically significant decrease in both the percentage of participants who were at risk of depression and in the depressive symptoms reported compared to baseline values [35]. Furthermore, in response to the open-ended questions, participants described the positive effect that being part of the program had on their mental health, attitude towards life, sense of purpose, and motivation to get out of the house [35]. Participants also mentioned that the program was a good opportunity to meet and connect with other people [35]. COV also measured depressive symptoms using both the Patient-Health Questionnaire-8 and an NIH toolbox for sadness, anxiety, and loneliness [38]. Although there were no statistically significant differences between groups at 6 months, participants in both the IG and CG reported a decrease in depressive symptoms, sadness, anxiety, and loneliness [38]. In addition, COV also measured cognitive outcomes through the Trail Making Test, which evaluates memory and executive function [38]. There were no significant differences in the changes of these cognitive outcomes when comparing the intervention and control groups [38].

HLWP also assessed participants’ moods and feelings, using a five-point Likert scale developed for the study [9] and found no significant pre-post changes. However, in response to open-ended questions at the end of the program, participants reported having enjoyed the intervention, seeing their friends, and learning together how to be healthier. Participants also reported high compliance with self-management activities [9].

Similarly to COV, BAILAMOS^TM^ used a combination of validated neuropsychological tests to measure participants’ cognitive performance in three domains: executive function, working memory, and episodic memory [39]. Participants improved their scores for global cognition, executive function, and episodic memory, although there were no statistically significant differences between IG and CG [39]. The study also used a qualitative component (group discussions) for participants to share their experiences and the benefits they gained from attending the program. Responses included having more energy throughout the day, more motivation, and valuing the opportunity to make new friends, among others [39].

#### 3.4.5. Behavioral Theory Related Outcomes and Results

Only two studies explicitly examined the effect that the intervention had on behavioral constructs: Let’s Walk! and Worth the Walk [36,37]. Let’s Walk! assessed participants’ self-efficacy related to regularly engaging in exercise through a modified version of the Lorig scale, which was translated to Spanish [36]. The study also evaluated individuals’ outcome expectations for exercise, using the Outcome Expectation for Exercise Scale [36]. Lastly, it also assessed age expectations in older adults through the ERA-12 tool (which considers expectations around general health, mental health, and cognitive function) [36]. At the end of the dancing program, participants in the IG had higher scores for self-efficacy than participants in the CG, but the difference was not statistically significant [36]. There were likewise no statistically significant differences between groups in outcome expectations for exercise [36]. Conversely, the increase in positive expectations related to aging and mental health among those who received the dance lessons was statistically significant at the 12-month time point. However, no other statistically significant differences between groups were found for the other expectations regarding aging [36].

Similarly, Worth the Walk assessed outcome expectations for exercise, stroke preparedness, and self-efficacy for managing stroke risk factors [37]. Participants in the IG reported better stroke preparedness and higher self-efficacy at the end of the program than participants in the CG [37]. These differences were statistically significant. In contrast, changes in outcome expectations for exercise were not statistically significant between the groups [37].

### 3.5. Latino Cultural Elements

Table 5 summarizes the strengths, limitations, and culturally relevant characteristics for Latinos in each study. Six of the studies (from four interventions) included in this review were tailored to Latino populations alone (COASH, AEA, Let’s Walk!, BAILAMOS^TM^ and BAILA) [15,34,35,36,39,40], and three included other ethnic minorities (HLWP, Worth the Walk, and COV) [9,37,38]. A wide variety of intervention characteristics made them relevant to Latinos in terms of setting, delivery, and/or content.

COASH study was developed in Mexico by Mexican health professionals and for Mexican older adults. As such, all the program components were developed and delivered in Spanish. The intervention was incorporated into an existing health center in Mexico City, which already provided social and cultural activities to individuals affiliated to the IMSS. Because of this, COASH organically integrated social, cultural, and environmental characteristics into its components. These characteristics make it very relevant to the intended beneficiaries [34].

Of the programs and interventions conducted in the US, all were offered in English and Spanish and were delivered by either bilingual or bicultural individuals [9,15,35,36,37,39,40]. The exception might be COV, which specifies that only some of the professional choir directors and accompanists who delivered the choir sessions were bilingual [38].

HLWP considered the role that the built environment plays in lifestyle choices of aging populations of minority groups [9]. Therefore, the senior housing community centers in which the program was implemented were located in an area with the highest percentage of minority older adults living at or below the poverty line [9].

Another culturally relevant aspect of the interventions was the reliance on individuals from the community who were trained to deliver the intervention and conduct the sessions/workshops. These individuals included facilitators working at the settings in which the intervention took place (COASH, HLWP and Worth the Walk) [9,34,37], *promotoras* known within the community (AEA) [15,35], Latino dance instructors (BAILAMOS^TM^ and BAILA) [39,40], choir directors from local communities (COV) [38], participants who were then trained as instructors (BAILA) [40], and individuals recruited from the general community who were trained to become health educators and facilitators (Let’s Walk!) [36].

Delivery of the programs also included culturally relevant aspects. For example, in Let’s Walk! family members or close friends of the participants could attend the educational sessions without enrolling in the study [36]. In this way, intervention delivery aligned with the value placed on *familia* and social connections within the Latino community [36,53,54]. Furthermore, the IG in this trial received some content in a culturally appealing format: *fotonovelas*, which is an effective communication strategy for Latinos [36,55,56]. The dramatized short story depicted an older Latino woman as a protagonist [36].

Five studies (from four interventions) explicitly incorporated elements that are culturally relevant for Latinos into their curriculum content: AEA, Worth the Walk, COV, BAILAMOS^TM^, and BAILA [15,35,37,38,39,40]. 

AEA fully integrated two key Latino cultural values into the intervention: the role of Catholicism and faith, and women’s caregiving roles and responsibilities within a family. Furthermore, the intervention was delivered in a church setting, and the cultural congruence of this setting may have helped foster engagement and motivation in participants who enrolled [15,35]. This was made evident in the qualitative component at the end of the intervention, in which participants mentioned having valued the inclusion of faith and having found Saints’ stories and parables motivating to become healthier [35]. Participants were allowed to change group allocation, and five chose to be assigned to the religious group [15]. The authors note that this group had the least dropouts [15,35]. Regarding the intergenerational content of the intervention, participants valued learning about activities they could do with their grandchildren at home, although the time spent caring for them sometimes prevented some participants from attending the program [15].

Worth the Walk also mentions that some sessions were culturally tailored based on insight gained during formative work and recommendations from the Latino mini-community advisory committee [37]. The two culturally tailored sessions framed walking around family and health, two relevant Latino values. The names of those sessions were ‘Walking if Good for Health and Relieving Stress’, and ‘Family Matters’. Sessions were different (and had different names) for the three other ethnic groups that were part of the study [37].

The choir (COV) and dancing interventions (BAILAMOS^TM^, BAILA) both described how the music, songs, and dance styles selected for the intervention were culturally relevant for Latinos [38,39,40]. For COV, choir directors identified music repertoires that could be culturally tailored for each site [38,57]. Moreover, as the intervention progressed, participants from each site were asked about their favorite music and provided suggestions about songs or arrangements [57]. Because this was a multi-cultural intervention, the music selection in ethnically diverse sites incorporated songs from various cultures, sometimes in different languages [57].

BAILAMOS^TM^ also included culturally relevant content in its curriculum [39,40]. Authors were thorough in their formative work to help ensure the cultural appropriateness of the intervention [39]. The dance program then incorporated input from focus group discussions with potential participants, community members, and stakeholders from Latino heritage to further understand their needs and preferences related to the intervention [39,40]. During this phase of the study, a bilingual Latino dance instructor was mentioned who was then contacted and who helped co-create the dance program [39,40]. Dance styles taught during the program were merengue, cha-cha-cha, bachata, and salsa (which were mentioned during formative work) [39,40]. Additionally, the program also incorporated *fiestas de baile* [dance parties], as opportunities for participants to socialize and practice the dance styles they had been learning [39,40].

## 4. Discussion

This scoping review aimed to investigate the effectiveness of theory-based interventions among aging community-dwelling Latino adults (≥50) on PA and nutrition related outcomes. The studies included in this review described multi-component theory-based interventions primarily promoting healthy aging behaviors around PA and diet [9,15,34,35,36,37,38,39,40]. However, most of them also assessed the effect of interventions on a variety of psychosocial and well-being outcomes [9,15,34,35,36,37,38,39], including anthropometric indicators and biomarkers (HLWP and Worth the Walk) [9,37].

All interventions in this review included multiple components that were delivered in person, mostly in group settings [9,15,34,35,36,37,38,39,40]. The programs and interventions described in this review varied in terms of aims, content, duration, and delivery format [9,15,34,35,36,37,38,39,40]. However, it is noteworthy that all included multiple components that, together, provide evidence for a holistic approach to promote older adults’ well-being [9,15,34,35,36,37,38,39,40]. This was further demonstrated by the inclusion in all of the studies of one or more tools to evaluate emotional well-being or perceptions around quality of life [9,15,34,35,36,37,38,39,40]. These characteristics align with the goals of the Decade of Healthy Aging in the Americas [16] and such an integrative and holistic approach has been recommended as an effort to promote healthy aging in all individuals [16,58]. Furthermore, combining different indicators to assess healthy aging also allows researchers to capture individual variability in aging rates using both objective and subjective factors [59].

Previous reviews have found similar results in terms of multi-component studies. For example, a systematic review of nutritional interventions for the management of frailty in older adults conducted by De Moraes et al. [29] found that most of the studies combined a nutrition education component with other elements [29]. Another systematic review by Floegel et al. examined PA and exercise interventions for older adults with heart failure [26]. The authors similarly found that some of the interventions also included health education sessions [26]. A systematic review and meta-analysis by Neves et al. also found that interventions seeking to improve nutritional outcomes among older adults often included a PA or health component [18].

The benefits associated with being active during the aging process are well known. However, older adults might experience some barriers to being physically active, such as lack of motivation, fear or stress around falls, and development of frailty [4,60]. Our present review found that the participants enrolled in these interventions successfully engaged in different types of PA. Overall, these studies showed changes, although not always statistically significant, in healthy aging behaviors, particularly related to active aging. Across the different outcomes reported in these studies (e.g., daily and weekly steps, gait, balance, time spent being physically active), PA measures changed in the expected direction. The exception might be COV, in which there was an inconsistent change in participants’ strength, balance, and gait speed outcomes between IG and CG and across different timepoints [38]. Statistically significant increases in PA were found in COASH, Let’s Walk!, Worth the Walk, BAILAMOS^TM^, and BAILA [34,36,37,39,40]. In addition, studies that incorporated a qualitative component found that participants felt more motivated to walk and be physically active [9,35]. Of note, some of the questionnaires used to assess PA-related outcomes asked participants about the time they spent in specific activities or locations, which could miss other PA opportunities that could have been influenced by the intervention [34,38,39,40]. Moreover, some of these PA-related outcomes were self-reported, and PA might have been overestimated or inactive time underestimated [61]. For example, in BAILAMOS^TM^ self-reported increases in PA were statistically significant but this was not the case for device-measured PA, although the opposite was found in Let’s Walk! [36,39]. Nonetheless, these results suggest that interventions of varying duration promote specific (i.e., walking, as in Let’s Walk! And Worth the Walk [36,37]) or culturally relevant activities (i.e., dancing Latino dance styles, as in BAILAMOS^TM^, and BAILA [39,40]) have the potential to increase PA in aging Latino adults. Notably, results from these interventions reported statistically significant changes in other well-being outcomes, such as improvement in health-related quality of life (COASH) [34], decreases in depressive symptoms (AEA) [35], and more positive expectations around aging (Let’s Walk!) [36]. Similarly, HLWP, COV, and BAILAMOS^TM^ [9,38,39] described positive effects, although not statistically significant, on well-being outcomes. This was further confirmed through the qualitative evaluations included in some of these studies, during which participants shared the benefits that the interventions had on their overall energy, mental health, and motivation [9,15,35,39]. Some specific components that might influence these positive outcomes include the in-person regular group meetings or workshops in familiar settings, activity modeling, incorporation of culturally relevant content or delivery strategies, opportunities for social interaction, clear incorporation of theoretical underpinnings, and implementation of intervention by a trained and trusted facilitator. Having interventions delivered by someone trusted by the community might help reduce some of the barriers that aging adults face to being physically active [49]. In the present review, studies reported different training sessions with intervention implementors, who were *promotoras*, community members, community health workers, Latino dance instructors, and even buddies to provide support for each other [9,15,34,35,36,37,38]. In BAILAMOS^TM^, participants shared positive thoughts about the Latino instructor, who was a key motivator for them to engage in dancing and PA [39]. Beyond being bilingual, future interventions should consider the identity and background of the individuals who will deliver the intervention, as well as the level of trust by the intended audience.

Considering nutrition, not all studies included in the present review reported the effect of the intervention on food intake or other diet-related outcomes. Statistically significant results were found in AEA, in which participants had favorable changes in the number of meals per day, the number of days per week in which fruit was consumed, and in the weekly consumption of fried foods [15,35]. Statistically significant changes were also found in HLWP, as the authors reported decreases in BMI, waist circumference, and blood glucose (which was self-reported) [9]. Other studies found positive, although not statistically significant, improvements in nutrition and diet-related outcomes, such as COASH and AEA [15,34,35]. As was previously mentioned, in the qualitative component included in some of the studies, participants perceived an overall improvement in their health and diet quality as a result of the interventions (AEA, HLWP, and BAILAMOS^TM^) [9,15,35,39].

These positive results are consistent with the previous literature. A review conducted by Floegel et al. also found that PA and exercise interventions have a positive and beneficial effect on participants’ quality of life and other outcomes (such as medication use) [26]. Similarly, a review by Wright et al. [31] looked at the effect that oral nutrition support with and without exercise had on older adults with sarcopenia, cachexia, or who were at risk of malnutrition. The authors found that participants who received an intervention combining nutrition and exercise had greater benefits on some outcomes (such as muscle strength) compared to participants who only received nutrition interventions [31]. While similar results were not found in other outcomes, the authors from these reviews emphasize the importance of combining nutrition, PA, and health elements in interventions for older adults, as this promotes support for their overall well-being [26,31]. This is particularly relevant for community-dwelling adults, most of whom want to live independently as long as possible, and a healthy aging process increases the likelihood that this occurs [62].

Our findings related to mode of delivery, either in-person individual or group delivery, is also comparable to what was found by Arigo et al. [23]. Their review focused on interventions promoting PA, and their results found that face-to-face interventions (some with telephone support) were the most prevalent [23]. The authors also noted that promoting PA as an opportunity for social connection among older adults is an effective strategy to attend to the social needs of community-dwelling individuals who live alone [14,23,27,63]. Multiple-component interventions delivered in person are effective ways to promote healthy aging in older Latino adults by considering the many physical, social, and emotional needs that this population has [14,63,64]. Future interventions should keep at the forefront the many intertwined factors that determine a healthy aging process [14,60,64], as a multicomponent intervention can help address the various needs of older Latino adults, rather than only addressing one factor in isolation.

A challenge with multicomponent interventions lies in their implementation, particularly when there are multiple centers involved. In the present review, COASH, AEA, Let’s Walk!, and COV utilized robust process evaluation and ongoing efforts to ensure fidelity in the way that intervention was being delivered [34,35,36,38]. Along with training implementors, measuring intervention fidelity and implementation can help with the interpretation of the studies’ effectiveness on outcomes of interest [65].

On another note, partnering with community centers, clinics, and community members [9,15,34,35,36,37,38] might have contributed to some of the positive results from the included studies. Future interventions and programs should prioritize these partnerships, as they can be beneficial for recruitment, implementation, evaluation, and scale-up of the program. In this review, BAILAMOS^TM^, COV, and Worth the Walk included specific strategies to increase recruitment opportunities, as well as active efforts to increase retention of enrolled participants [38,39,41], both of which might be beneficial for future research. However, in future research it will be important to also consider the needs of older adults who, while independent community dwellers, may be more isolated and less connected to such centers since they are likely to be at higher risk of developing frailty syndrome or health problems [27].

All the studies in this review included a behavioral theoretical foundation (or relevant constructs that guided delivery strategies) and culturally relevant elements to make the interventions more appealing to the population of interest [9,15,34,35,36,37,38,39,40]. The use of theories and psychological constructs to develop interventions increased [23,28]. All the studies included in this review described, to different extents, the theoretical components that informed the content and format of the interventions [9,15,34,35,36,37,38,39,40]. However, only Let’s Walk! and Worth the Walk included specific measures to assess the intervention’s effect on theoretical constructs [36,37]. A scoping review by Arigo et al. found similar results related to the variety with which studies report, incorporate, and assess theoretical components [23]. Future interventions should include more detailed information about the behavioral theory underpinnings, such as explicitly describing how they are incorporated into the content or delivery strategies, as well as how behavioral constructs or components were assessed.

The need to culturally adapt behavioral interventions to the intended audience has been recognized as an element that enhances effectiveness and helps reduce disparities [66,67]. In the present review, some variation was observed when describing the culturally relevant components that were included. For example, AEA [15,35] detailed the formative research process that took place before the intervention, as well as the process of incorporating those findings into the intervention curriculum. It considered core Latino values (family and religion) throughout the intervention, including its name [15,35,45,46]. On the other hand, HLWP included fewer details about culturally relevant elements beyond the sessions being delivered in English and Spanish [9]. Future studies among Latino older adults (or other populations) should make cultural adaptation a priority and consider how to tailor all phases and aspects of the intervention to their intended audience. Using frameworks can be helpful in guiding and documenting this process [68].

There was a high percentage of female participants in the studies included in this review. Prior reviews have found a more even distribution of participants’ gender [26], although a review by Wright and Baldwin similarly found a high enrollment of female participants, although not as high as in this review [31]. Some of these differences might be partially explained by the high value that Latino populations place on gender roles, which have been found to influence a person’s attitudes, perception of self-worth, family responsibilities, and health attention-seeking behaviors, among others [69,70]. In general, individuals identifying as male might be less likely to seek professional assistance to treat or prevent health problems [69,70]. Therefore, future research to promote healthy aging behaviors in male Latino adults should consider these cultural characteristics in the design, recruitment, and implementation of the interventions.

The present review has some limitations. It only included nine studies that assessed the effects of seven distinct interventions. Given the small number of studies and their noticeably varying characteristics, clear themes did not emerge related to the factors that could enhance or hinder the effectiveness of interventions delivered among aging Latinos. Another limitation is that the screening, extraction, and synthesis of studies were conducted by only one author, which opens the possibility of individual biases affecting the selection and analysis of studies. This was mitigated by working with a reference librarian during the development of the initial search strategy and by carefully applying the inclusion/exclusion criteria during the study selection process. Furthermore, the authors met periodically to discuss the scope of the work, inclusion of studies (until agreement was reached, if there was uncertainty), and relevance and interpretation of findings. This study has some strengths. Despite the small number of studies that were included, results show promising, although not always statistically significant, trends on the effect that theory-based programs with culturally relevant elements have on healthy aging behaviors in Latinos. This review also provides some insights for future intervention developers who work with Latino older adults. Our findings can help researchers identify key characteristics to focus on when designing and implementing culturally relevant interventions to increase their effectiveness on promoting healthy aging among older Latinos.

## 5. Conclusions

This scoping review contributes to the growing body of literature that assesses the use and effectiveness of theory-based interventions to promote healthy behaviors in aging populations. Compared to prior reviews, the present study focused on interventions that targeted community-dwelling Latino aging adults (≥50). Therefore, it also discussed the culturally relevant characteristics used when delivering health education sessions for this population. Researchers should consider how different ethnicities define a healthy aging process when determining the behaviors and outcomes to be encouraged. Future behavioral interventions should integrate a planned, purposeful, and rigorous cultural adaptation process into their theoretical foundations to ensure their relevance and effectiveness for Latino older adults. More careful documentation and description of the behavior change theoretical foundation and the cultural adaptation process could also facilitate implementation, replication, and scale-up of effective interventions for Latinos.

## Figures and Tables

**Table 1:** Search strategy for identifying relevant studies.

Database	Search String
Web of Science	TI = ((diet* OR nutrition* OR “well-being” OR “lifestyle*” OR “eat*” OR “health*” OR “food choice*” OR “activ*” OR “movement” OR “sedentar*” OR “exercise” OR “physical activity*”) NEAR/3 (treatment* OR therap* OR promot* OR education* OR intervention* OR modif* OR change* OR program*)) AND TO = (“older adult*” OR “senior*” OR “elder*” OR “geriatr*” OR “veteran*” OR “age” OR “aging” OR “retired”) AND TO = (“Hispanic*” OR “Latin*” OR “chican*” OR “Mexican-American” OR “Caribbean” OR “Central American” OR “Non Caribbean” OR “minorit*” OR “ethnic minorit*” OR “vulnerable*”) AND TO = (“theor*” OR “framework” OR “behavior* change*” OR “model” OR “construct*”)
PubMed	(“diet” [Title/Abstract] OR “nutrition” [Title/Abstract] OR “well-being” [Title/Abstract] OR “lifestyle” [Title/Abstract] OR “eat” [Title/Abstract] OR “health” [Title/Abstract] OR “food choice” [Title/Abstract] OR “active” [Title/Abstract] OR “movement” [Title/Abstract] OR “sedentary” [Title/Abstract] OR “exercise” [Title/Abstract] OR “physical activity” [All Fields]) AND (“treatment” [Title/Abstract] OR “therapy” [Title/Abstract] OR “promotion” [Title/Abstract] OR “education” [Title/Abstract] OR “intervention” [Title/Abstract] OR “modification” [Title/Abstract] OR “change” [Title/Abstract] OR “program” [Title/Abstract]) AND (“older adult” [Title/Abstract] OR “senior” [Title/Abstract] OR “elder” [Title/Abstract] OR “geriatric” [Title/Abstract] OR “veteran” [Title/Abstract] OR “age” [Title/Abstract] OR “aging” [Title/Abstract] OR “retired” [Title/Abstract]) AND (“hispanic” [Title/Abstract] OR “latin” [Title/Abstract] OR “chicano” [Title/Abstract] OR “Mexican-American” [Title/Abstract] OR “Caribbean” [Title/Abstract] OR “Central American” [Title/Abstract] OR “Non Caribbean” [Title/Abstract] OR “minority” [Title/Abstract] OR “vulnerable” [Title/Abstract]) AND (“theory” [Title/Abstract] OR “framework” [Title/Abstract] OR “behavior change” [Title/Abstract] OR “model” [Title/Abstract] OR “construct” [Title/Abstract]) Filters applied: Full text, Clinical Trial, Meta-Analysis, Randomized Controlled Trial, Review, Systematic Review, Humans, English, Spanish, ged + Aged: 45+ years, Middle Aged: 45-64 years, Aged: 65+ years, 80 and over: 80+ years.

* For Web of Science, we included an asterisk (*) in some of our search terms to find variant spellings of those terms.

**Table 2:** Studies’ and participants’ characteristics.

Citation	Intervention Name	Study Design	Duration and Follow-Up	Sample Size and Participants Characteristics	Inclusion Criteria	Aim
Pérez-Cuevas et al. (2015) [34]	Centre of Social Health Services for Older Adults, (COASH) pilot program	Pre-post evaluation of pilot program, no control group	1 year No follow-up	n = 239 Median age = 77 Female = 67.8% Diagnosis of chronic diseases = 97.5%	Mexican older adults (>65) affiliated to the Mexican Institute of Social Security (IMSS), with mild-to-moderate physical dependency, no falls or injuries within the past 72 h.	To design and evaluate a pilot program aimed at promoting active aging in older adults at IMSS.
Schwingel et al. (2015 and 2017) [15,35]	*Abuelas en Acción* (AEA) [Grandmothers in Action]	Quasi-experimental mixed-methods study, no control group	9 months 6 months of intervention + 3 months of maintenance	n = 34 Mean age = 64.3	Women (≥50), self-identified as Latino, members of the local community.	To develop, implement, and evaluate an evidence-based lifestyle intervention addressing PA, nutrition, and stress management in older Latino women.
Tallier et al. (2017) [9]	Healthy Living Wellness Program (HLWP)	Pretest/posttest quasi-experimental study, no control group	8 weeks No follow-up	n = 30 Mean age = 72.3 Female = 70% Latino = 47%	Convenience sample from an area with the highest percentage of minority older adults (≥65) living at or below the poverty line, with the ability to speak and read English or Spanish.	Examine the effects of HLWP on the adaptation of health status including BMI, waist circumference, body weight, blood pressure, blood glucose, mood/feeling, and PA among minority underserved and economically disadvantaged older adults.
Piedra et al. (2018) [36]	Let’s Walk! [¡*Caminemos*!]	Double-blind RCT	2 years 4 weeks of intervention 12-month follow-up 24-month follow-up	n = 572 Mean age = 73.1 Female = 77.1%	Adults (≥60), self-identified as Latino, fluent in English or Spanish, cognitively intact, able to walk (use of canes or walkers was permitted), physically inactive (<20 min of exercise at least 3 times/week), available to attend exercise and education classes.	Determine whether a random sample of older Latino adults exposed to the Let’s walk! curriculum would experience an enhanced response to a modified version of a low-cost exercise program (EnhanceFitness^®^) when compared to those who received a health education curriculum.
Menkin et al. (2019) [37]	Worth the Walk	Single-blind, randomized wait-list controlled trial	3 months 1 month of intervention 3-month follow-up	n = 233 Mean age = 73.9 Female = 69.1% Latino = 27%	Adults (>60), with self-reported hypertension, with ability to walk (assistive devices allowed) and to sit in a class setting; self-identified as racial/ethnic groups, able to communicate in specific languages, and available to attend all sessions.	To test the effectiveness of a potentially sustainable, culturally tailored intervention to increase walking, stroke knowledge, self-efficacy, positive beliefs about exercise, quality of life and clinical health indicators among Latino, Korean, Chinese, and White seniors.
Johnson et al. (2020) [38]	Community of Voices (COV), [*Comunidad de Voces*]	Cluster randomized waitlist-controlled trial	1 year No follow-up	n = 390 Mean age = 71.1 Female = 76.5% Latino = 18%	Adults (>60) having sufficient visual and hearing acuity (with assistive devices), and being fluent in English or Spanish (bilingual and monolingual speakers).	To test effects of the COV choir intervention on health, well-being, and healthcare costs of racial/ethnically diverse older adults.
Balbim et al. (2022) [39] and Marquez et al. (2022) [40]	BAILAMOS^TM^ [We are dancing] Pilot Study BAILA [Dance] trial	BAILAMOS^TM^ Pilot Study Parallel, two-armed randomized controlled mixed methods pilot study BAILA trial Two-condition RCT	BAILAMOS^TM^ Pilot Study 4 months BAILA trial 8 months 4 months of dance program 4 months of maintenance	BAILAMOS^TM^ Pilot Study n = 57 Mean age = 65.2 Female = 83.9% BAILA trial n = 333 Mean age = 64.9 Female = 84.3%	Adults (≥55), self-identified as Latino, ability to understand Spanish, <2 days per week of aerobic exercise, at risk for disability, cognitively healthy, danced <2 times/month over the past year, willingness to be randomly assigned to a control group, no current plans to leave the US for two or more consecutive weeks.	BAILAMOS^TM^ Pilot Study To investigate the effects of the BAILAMOS^TM^ dance program on PA, cardiorespiratory fitness, and cognitive health. BAILA trial To describe self-reported and device-assessed changes in PA as a result of an RCT based on BAILAMOS^TM^, and a 4-month maintenance period, versus a health education control group.

RCT = Randomized Controlled Trial; BMI = Body Mass Index.

**Table 4:** Studies’ outcomes, measurements and results.

Intervention Name	Main Outcomes and Measurements	Key Results
Centre of Social Health Services for Older Adults, (COASH) pilot program [34]	- Regular PA: Self-reported compliance with PA guidelines (150 min/week during previous three months). Percentage of participants who comply. - Gait and balance: Modified Performance Oriented Assessment of Mobility Problems of Tinetti. Higher values = lower risk of falls (out of 28). - Basic activities of daily living (lying down, walking within house, dressing, bathing, eating, etc.): Barthel index. Higher values = more independence in basic activities (out of 100). - Instrumental activities of daily living (using the telephone, shopping, preparing food, handling finances, etc.): Lawton and Brody scales. Higher value = more independence in instrumental activities (out of 16). - Occupational functioning (volition, habituation, communication and interaction skills, process skills, motor skills, and environmental): Model of Human Occupation Screening Tool. Higher scores = better occupational functioning (out of 96). - Health-related quality of life (physical and psychological health, social relationships, environment, etc.): Measured with WHOQOL-BREF. Higher score = better perception of health-related quality of life.	Mean changes between endpoint and baseline: - Regular PA: Statistically significant increase of 25.8%. Adherence of >80% to the program associated with significant change. - Gait and balance: Statistically significant improvement of 0.4. Adherence of >80% to the program not associated with significant change. - Basic activities: Not statistically significant reduction of 0.3 points. - Instrumental activities: Not statistically significant improvement of 0.04 points. - Occupational functioning: Statistically significant improvement of 6.2 points. Adherence of >80% to the program associated with significant change. - Health-related quality of life: Statistically significant improvement of 2 points. Adherence of >80% to the program associated with significant change.
*Abuelas en* *Acción* (AEA) [Grandmothers in Action] [15,31]	- PA: Minutes of moderate-to-vigorous-PA (MVPA) from accelerometer data. - Food intake and nutrition outcomes: Measured using a food frequency questionnaire and a 24 h recall. - Depressive symptoms: Measured using a Spanish translation of the Center for Epidemiological Studies–Depression Boston 10 Form. - Open ended questions exploring perceptions about the program, with specific questions about each component.	QUANTITATIVE RESULTS: - PA: * Not statistically significant: (A) Increase of 48 MVPA min/week from baseline to 6 months, and of 20.4 min/week from 6-month to 9-month timepoints. (B) Increase in minutes of activity bouts by 12.6 min/week from baseline to 6 months, and of 9.5 min/week from baseline to 9 months. (C) Increase in percentage of participants classified as active (58.6% at baseline, 79% at 6 months, and 85.7% at 9 months). - Food intake and nutrition outcomes: * Not statistically significant: (A) Increase of 0.4 meals/day from baseline to 6 months, and increase of 0.1 from baseline to 9 months. (B) Decrease in hours between breakfast and lunch (4.2 at baseline, 3.7 at 6 months, and 3.9 at 9 months). (C) Decrease in hours between lunch and dinner (5.4 at baseline, 4.7 at 6 months, and 4.8 at 9 months). (D) Increase of 0.1 vegetables consumed per day from baseline to 6 months, and decrease of 0.1 vegetables per day from baseline to 9 months. E) Increase of 0.3 fruits consumed per day from baseline to 6 months, and decrease of 0.1 fruits per day from baseline to 9 months. * Statistically significant: (A) Increase in percentage of participants who ate three meals per day (59% at baseline, 89% at 6 months, and 85.7% at 9 months). (B) Increase in number of days/week that fruits are consumed (5.3 at baseline, 6.2 at 6 months, and 6.4 at 9 months). (C) Decrease in number of fried foods consumed per day (2.9 at baseline, 1.7 at 6 months, and 2.2 at 9 months). - Emotional well-being: * Statistically significant: A) Decrease of 0.1 score for depressive symptoms from baseline to 6 months, and of 3.3 from baseline to 9 months. B) Decrease in percentage of participants at risk of depression (100% at baseline, 78% at 6 months, and 53% at 9 months). QUALITATIVE RESULTS (At the end of program): - PA: The program motivated participants to walk more; interest in resistance training, walking as an appealing activity. - Nutrition outcomes: High awareness of healthy dietary behaviors, including reading nutrition labels. Focus on food variety. More confidence in talking about dietary health. - Emotional well-being: Recognition of program’s positive effect on mental health, more positive attitude toward life, opportunity to connect with others. - Religious content: Value of including faith into the program, parables, and Saints’ stories, which was motivating. - Intergenerational content: Reflection on time spent caring for grandchildren, which reduced attendance. Bringing children was distracting, although participants mentioned doing activities at home together.
Healthy Living Wellness Program (HLWP) [9]	- PA: Self-reported number of activity minutes per week (including the intervention weekly exercise). - Weight and BMI: measured with a calibrated electronic scale and wall = attached measuring stick. - Waist circumference: measured with a tape measure. - Blood pressure: measured using an electronic blood pressure monitor. - Blood glucose: Self-reported (participants used their home glucometers). - Mood/feelings: Five-point Likert-type scale developed for the study (1 = very sad, and 5 = very happy). - Open-ended questions: Benefits from the study and compliance with medication, daily exercise, adherence to medical appointments, and adherence to recommended diet modifications.	QUANTITATIVE RESULTS: - PA: Increase in minutes of PA [not specified]. - Weight: Not statistically significant decrease of 1.72 pounds after intervention. - BMI: Statistically significant decrease of 0.46 units after intervention. - Waist circumference: Statistically significant decrease of 0.5 in at post intervention. - Blood pressure: Not statistically significant decrease of 3.5 mmHg in Systolic pressure, and 3.73 mmHg in Diastolic pressure after intervention. - Blood glucose: Statistically significant decrease of 18.97 mg/dL between pre-post measurements. - Mood/feelings: Not statistically significant decrease of 0.3 in mean score after intervention. OPEN-ENDED QUESTIONS: - Compliance with self-management activities was reported as follows: 73% exercising daily, 90% attending healthcare appointments, 83% eating healthy, 97% general benefits from the program. - Benefits included awareness of medical conditions, understanding of the importance of adherence to medical treatment, regular exercise with other members of the group, enjoyment of group meetings and learning about being healthier.
Let’s walk! [¡*Caminemos*!] [32]	- PA: * Number of weekly steps, determined through a pedometer. * Self-reported perceptions of PA, using the Spanish version of the Yale Physical Activity Survey (YPAS). This generates three scores: (A) Total time spent on activities in a typical week during the past month; (B) For energy expenditure summary index, frequency and duration of PA in five dimensions (vigorous activity, leisure walking, moving, standing and sitting); and (C) Activity dimensions summary score (considering time spent and intensity of each of the five activity dimensions). - Expectations regarding aging: Using ERA-12 which measures age expectations in older adults, representing three domains: general health, mental health and cognitive function. Out of a score of 100, higher values = higher aging expectations. - Self-efficacy: Using a modified version of the Lorig scale, translated to Spanish. Higher values = more confidence in a person’s ability to regularly engage in exercise. - Outcome expectation for exercise: Measured with the OEE scale for older adults, scored from 1 to 5. Higher values = stronger outcome expectations.	- PA: * Statistically significant difference between groups at 1 and 12 months: Number of weekly steps: At 1, 12, and 24 months, IG had on average 844.5, 1198.9, and 1009.9 more weekly steps than the CG. * Not statistically significant: Self-reported perceptions of PA, considering YPAS scores: (A) At 1, 12, and 24 months, IG spent 0.6, 0.1, and 0.6 more hours/week doing different activities than the CG. (B) At 1, 12, and 24 months, IG spend 140.4, 37.1, and 219.7 more kilocalories/week than the CG. (C) At 1, 12, and 24 months, IG had 2, 3.5, and 2.5 higher points in the score of activity dimensions than the CG. - Expectations regarding aging: At 1, 12, and 24 months, IG had 1.3, 4.2, and 2.6 higher points than the CG. Not statistically significant. Statistically significant differences were found between the groups at 12 months for mental health score, with a difference of 8.8 points between groups. - Self-efficacy: Not statistically significant: At 1, 12, and 24 months, IG had +0.1, −0.1, and +0.2 points than CG. - Outcome expectations for exercise: At all time points, there were no differences in outcome expectations between groups.
Worth the Walk [37]	- Mean Daily Steps: measured with a pedometer. - Clinical health outcomes: * BMI: height (cm) and weight (kg) measured in duplicate at each time point. * Biomarkers: Using capillary blood samples to measure cholesterol, C-reactive protein and glycated hemoglobin. * Blood pressure: using an automated Omron device, following standard protocol with 5 min rest period between each measure). - Self-reported health outcomes: * Adapted Stroke Action test. * Adapted self-efficacy scale (higher value, higher confidence in managing stroke risk). * Outcome expectations for exercise (agreement with positive statements).	- Mean daily steps: * Not statistically significant: Pre-post change in mean daily steps increased 489 at 1 month, and 233 at 3 months in IG. * Statistically significant at 1 month [*p* < 0.01], yet not clinically significant: Being in the IG was associated with having 887 more daily steps at 1 month, and 947 more daily steps at 3 months than being in the CG. - Clinical health outcomes: * BMI: Not statistically significant: (A) Pre-post change in BMI decreased 0.02 units at 1 month, and 0.14 at 3 month in IG. (B) Being in the IG was associated with having 0.06 less units in BMI at 1 month, and 0.13 less units at 3 months than being in the CG. * Biomarkers (only baseline and second timepoint): Not statistically significant: (A) Pre-post change in non-HDL cholesterol increased 1.7 mg/dL at 3 month in IG. (B) Being in the IG was associated with having +11.7 mg/dL in non-HDL cholesterol at 3 months than being in the CG. (C) Pre-post change in HbA1c decreased 0.10% at 3 month in IG. D) Being in the IG was associated with having −0.12% in HbA1c at 3 months than being in the CG. * Blood Pressure: Not statistically significant: (A) Pre-post change in systolic blood pressure decreased 1.2 mmHg at 1 month, and 1.7 mmHg at 3 month in IG. (B) Pre-post change in diastolic blood pressure decreased 0.7 mmHg at 1 month, and 1.2 mmHg at 3 month in IG. (C) Being in the IG was associated with having +1.5 mmHg in systolic blood pressure at 1 month, and +2.1 at 3 months than being in the CG. (D) Being in the IG was associated with having +1.4 mmHg in diastolic blood pressure at 1 month, and +1.43 at 3 months than being in the CG. - Self-reported health outcomes: * Statistically significant: (A) Pre-post change in percentage of participants who reported stroke preparedness increased 19 pp at 1 month, and 16pp at 3 month in IG. (B) Being in the IG was associated with increasing 0.22 pp in stroke preparedness at 1 month, and 0.20 pp at 3 months than being in the CG. (C) Pre-post change in self-efficacy increased 0.3 points at 1 month, and 0.23 points at 3 month in IG. (D) Being in the IG was associated with increasing 0.37 points in self-efficacy at 1 month, and 0.59 points at 3 months than being in the CG. Three-month results are statistically significant. * Outcome expectations: Not affected by intervention.
Community of Voices (COV), [*Comunidad de Voces*] [38]	- Physical outcomes: Three performance-based measures were used to assess lower body strength, balance, and walking speed, measured through time in seconds it takes to complete five chair stands, the NIH Toolbox Standing Balance measure, and speed at walking 4 m (meters/second). - Psychosocial outcomes: Determined by depressive symptoms, assessed using the Patient-Health Questionnaire (PHQ-8). Scores range from 0 to 24 and higher scores indicate more depression. Other secondary psychosocial outcomes included sadness, anxiety, and loneliness (measures drawn and assessed with NIH Toolbox). - Cognitive outcomes: Determined by the Trail Making Test (TMT), which measures memory and executive function. Measured in seconds it takes to complete. Other secondary cognitive outcomes included attention and inhibitory controlled (measured with a modified version of the NIH).	- Physical outcomes: * Not statistically significant: (A) Pre-post change showed an increase of 0.4 s to complete five chair stands in the IG, and a decrease of 0.3 s in the CG. (B) Group-by-time differences at 6 months. (C) Differences found in balance nor gait between IG and CG. - Psychosocial outcomes: * Pre-post change showed a decrease of 0.3 points in the PHQ-8 score in the IG, and a decrease of 0.1 points in the CG. Not statistically significant group-by-time differences at 6 months. - Cognitive outcomes: * Pre-post change showed an increase of 1.1 s in TMT in both the IG and in the CG. Not statistically significant group-by-time differences at 6 months.
BAILAMOS^TM^ [We are dancing] Pilot Study [39] BAILA [Dance] trial [40]	In BAILAMOS^TM^ Pilot Study - PA: PA level, and in leisure time, with an accelerometer and self-reported through the Community Health Activities Model Program for Seniors PA Questionnaire for Older Adults (CHAMPS). - Cardiorespiratory Fitness: Determined with a previously validated regression equation. - Cognition: Four neuropsychological tests measured attention and executive function: TMT, Stroop C of the Stroop Neuropsychological Screening Test and the Stroop color–word task, word fluency test, and Symbol Digit Modalities Test. - Discussions to evaluate the program: program length, frequency and duration of classes, dance instructor (e.g., quality of instruction, attention, and pace), music, and things that prevented or helped participants to attend classes. In BAILA trial: - PA: Assessed by the Community Healthy Activities Model Program for Seniors (CHAMPS) Physical Activity Questionnaire for Older Adults and by use of accelerometer.	In BAILAMOS^TM^ Pilot Study **QUANTITATIVE RESULTS:** - PA: Statistically significant increased self-reported participation in leisure-time moderate-to-vigorous PA in IG compared to CG, at post intervention. Non-statistically significant difference in device-assessed PA. - Cardiorespiratory Fitness: Was not affected. - Cognition: Non-statistically significant changes in cognitive domains, between IG and CG, although pre-post significant changes per group in global cognition, executive function and episodic memory. **QUALITATIVE RESULTS:** Participants thought classes were enough and appropriate; benefits that were mentioned included feeling more energized, with better motor coordination, more motivated, sense of discipline, new friendships, and impact on health. Appreciated instructor’s energy, attention, and ability to motivate. Found the music joyful. In BAILA trial: - PA: At the 4-month time point, total PA in IG increased by 192 min/week, and by 622.11 at 8 months after the maintenance phase compared to an increase of 165.94 min/week in the CG at 4 months, and of 66.85 min/week at the 8-month timepoint. These differences were statistically significant. Similar trends were observed for weekly minutes of moderate-to-vigorous PA, and leisure PA. Non-statistically significant differences were observed for PA at the household.

BMI = Body Mass Index; IG = Intervention Group; CG = Control Group.

**Table 5:** Studies’ strengths, limitations, and culturally relevant characteristics for Latinos.

Intervention Name	Strengths	Limitations	Culturally Sensitive Characteristics for LATINOS ^¥^
Centre of Social Health Services for Older Adults, (COASH) pilot program [34]	- Considered implementation and feasibility factors for scale up (integrating the program to services provided by IMSS). - Included individually tailored components based on comprehensive assessment. - Incorporated recruitment and retention strategies, and considered the effect that adherence to the program had on outcomes. - Intervention promoted a holistic perspective of well-being and quality of life. - Intervention was developed and implemented by a multi-disciplinary team, including a geriatrician, rehabilitation specialist, psychologist, social worker, dietitian, information technology educator, nurses, among others. - COASH personnel facilitating the program received training for one month before the pilot study and were reinforced periodically.	- No CG. - No follow up. - No mention of assessing implementation fidelity or program delivery. - Not all assessment tools had been validated in Mexican populations. - No assessment of health personnel’s thoughts on feasibility and acceptability of the program. - Participants not fully representative of all the older adults affiliated to IMSS.	* Developed: - In Mexico City; - By and for Mexican adults; - Within the Mexican health system; - In health centers that already provide social and cultural activities; - Sensitive and relevant to the social and cultural context. - Fully developed and conducted in Spanish.
*Abuelas en* *Acción* (AEA) [Grandmothers in Action] [15,31]	- Development of intervention was informed by formative work (which included photo-elicitation and individual interviews with older Latino women). - Community based participatory research, which involved partnership with a local Catholic church. - Training of *promotoras* about program components and delivery strategies. - Included maintenance phase. - Mixed-methods approach to evaluate the intervention. - Considered feasibility of implementation, sustainability and potential reach, using RE-AIM framework, and included interviews with participants, *promotoras*, community leaders, and priests at the end of the program. - Qualitative findings were translated and assessed through a translation/back-translation process.	- No CG. - Some participants reallocated to the religious group. - Small sample size. - High percentage of dropouts. - Program might not be relevant for older Latino women who do not attend religious services so frequently.	- Health promotion curriculum heavily incorporated culturally relevant topics: - Faith, religion and spirituality; - Family values, roles and caregiving responsibilities. - The name of the program considers the importance of family to Latinos. - Training, workshops, data collection and program delivery took place in Catholic church facilities within a Latino enclave. - Delivered by trained *promotoras* from the community, familiar with local customs and traditions. - Intervention, interviews and all materials available in Spanish and English. - Research team included native Spanish speakers.
Healthy Living Wellness Program (HLWP) [9]	- Partnership with senior housing community centers. - Training of registered nurses and interns working at the centers to deliver the program. - Included individual-level screening assessment. - Included teaching participants how to measure their own blood pressure and anthropometric measures. - Assessment of perceived benefits from participation in the program.	- Small sample size from a convenience sample. - No CG. - Short duration of intervention and no follow-up period. - No mention of assessing implementation fidelity or program delivery. - High missing values in some outcomes and reliance on self-reported data. - Lack of rigorous external and internal validity.	- Intervention sessions delivered in English and Spanish. - Senior housing community centers from an area with the highest percentage of minority older adults.
Let’s walk! [¡*Caminemos*!] [36]	- Double blinded study. - Community-based participatory research, including partnerships with different senior centers. - Trained community members to become facilitators. - Included a reinforcement phase and long follow-up period. - Included both self-reported (which has risk of recall bias) and objective measures. - Robust process evaluation (measuring fidelity of curriculum delivery). - Assessed levels of acculturation in participants. - Assessed behavioral theory constructs.	- Scheduling of sessions was decided by each site (which might compromise validity, but facilitates feasibility and acceptability by community partners). - Risk of group contamination (randomization was at individual level). - CG also received cognitive support and exposure to exercise classes. - Use of pedometers instead of accelerometers to estimate weekly steps.	- Allowed spouses/housemates to attend classes without enrolling in the study. - Intervention delivered in Spanish or English by trained facilitators from the community. - Delivery of information for the IG included the use of *fotonovelas* with a senior Latino protagonist.
Worth the Walk [37]	- Included follow-up (although somewhat short-term). - Community-partnered participatory research, and intervention was integrated into ongoing programming at senior centers. - Trained site case managers to deliver the intervention. - Included strategies to encourage retention. - Intervention development was informed by formative work. - Inclusive study promotes generalizability (yet authors suggest caution with cultural tailoring, as it is complex). - Assessed behavioral theory constructs.	- Short intervention. - Pedometer non-adherence and inconsistency in use was high. - Provision of the pedometer could also influence behavior, particularly in CG. - Authors cannot rule out the possibility of effect on daily steps reflecting regression to the mean.	- Delivered in Spanish or English by bilingual case managers from the senior sites. - Intervention included two culturally tailored sessions for Latinos, with insight from ethnic-specific community action boards and 12 focus groups. The two sessions incorporated cultural values of health and family: ‘Walking is Good for Health and Relieving Stress’ and ‘Family Matters’ - Survey instruments were forward- and back-translated into Spanish
Community of Voices (COV), [*Comunidad de Voces*] [38]	- Intervention delivered in senior centers to which participants frequently attended. - Included robust process evaluation with comprehensive fidelity checks to measure fidelity of delivering the choir intervention. - Training of choir directors and accompanists to deliver the intervention. - High retention rate and engagement of participants.	- Difficulties collecting data in Spanish and English. - High risk of group contamination within the centers.	- Selection of music style (Latin folk music) by senior center director and considering the background and preferences of older adults each center served. - Intervention available in English and Spanish at senior centers with which participants were already familiar. - Intervention sessions included discussions about the music pieces.
BAILAMOS^TM^ [We are dancing] Pilot Study [39] BAILA [Dance] trial [40]	In BAILAMOS^TM^ Pilot Study - Intervention was developed informed by formative work - Mixed methods approach. - Community based participatory research, with partnerships with senior centers. - Recruitment took places at churches and other places frequently attended by Latino older adults. In BAILA trial: - Inclusion of maintenance phase. - Included measures of acculturation and body composition. - Included indigenous dancers who became trainers for the maintenance phase. - Active strategies to reduce the risk of contamination and crossover in senior centers. - Included strategies for scale up and future low-cost implementation (i.e., trainers).	- Small sample size (although this was a pilot study). - Inconsistent use of device-assessed PA, increasing reliance on self-reported data. - Participants in CG received accelerometer, which might influence behavior. - Potential for contamination at centers.	- Recruitment also took place at a Catholic church near the senior center. - Focus on a culturally relevant PA (dancing). - Included music and dancing styles preferred by participants. - Intervention incorporated culturally relevant social events with food and drinks (*fiestas de baile*). - Intervention delivered by known Latino dance instructor, and available in English and Spanish. - The name of the program is a verb is Spanish. In BAILA trial: - Included empowering indigenous participants, who became trainers.

^¥^ These culturally relevant characteristics were not always explicitly mentioned in the studies but were put together by the authors. IG = Intervention Group; CG = Control Group.

## Data Availability

Not applicable.
